# Patient driven care in the management of prostate cancer: analysis of the United States military healthcare system

**DOI:** 10.1186/s12894-017-0247-x

**Published:** 2017-07-11

**Authors:** Muhammad Ali Chaudhary, Jeffrey J. Leow, Matthew Mossanen, Ritam Chowdhury, Wei Jiang, Peter A. Learn, Joel S. Weissman, Steven L. Chang

**Affiliations:** 1 Center for Surgery and Public Health, Department of Surgery, Brigham and Women’s Hospital, Harvard Medical School, Harvard T H Chan School of Public Health, Boston, MA USA; 2 Division of Urology, Brigham and Women’s Hospital, Harvard Medical School, Boston, MA USA; 3 Department of Surgery, Uniformed Services University of Health Sciences, Bethesda, MD USA

**Keywords:** TRICARE, Robotic surgery, Prostatectomy, Patient preference

## Abstract

**Background:**

Patient preferences are assumed to impact healthcare resource utilization, especially treatment options. There is limited data exploring this phenomenon. We sought to identify factors associated with patients transferring care for prostatectomy, from military to civilian facilities, and the receipt of minimally invasive radical prostatectomy (MIRP).

**Methods:**

Retrospective review of 2006-2010 TRICARE data identified men diagnosed with prostate cancer (ICD-9 185) receiving open radical prostatectomy (ORP; ICD-9: 60.5) or MIRP (ICD-9 60.5 + 54.21/17.42). Patients diagnosed at military facilities but underwent surgery at civilian facilities were defined as “transferring care”. Logistic regression models identified predictors of transferring care for patients diagnosed at military facilities. A secondary analysis identified the predictors of MIRP receipt at civilian facilities.

**Results:**

Of 1420 patients, 247 (17.4%) transferred care. These patients were more likely to undergo MIRP (OR = 7.83, *p* < 0.01), and get diagnosed at low-volume military facilities (OR = 6.10, *p* < 0.01). Our secondary analysis demonstrated that transferring care was strongly associated with undergoing MIRP (OR = 1.51, *p* = 0.04).

**Conclusions:**

Patient preferences induced a demand for greater utilization of MIRP and civilian facilities. Further work exploring factors driving these preferences and interventions tailoring them, based on evidence and cost considerations, is required.

## Background

The modern healthcare sector is complex, with supply and demand operating at patient, provider, and facility levels. Different factors play a vital role in creating and increasing the demand for utilization of certain healthcare services [1–4]. Among them the effects of hospital and provider level factors have been well described in the literature. Delamater and colleagues have shown that the hospitalization rate in a particular geographical area is directly related to the number of hospital beds available [1], pointing towards the role of hospitals in creating a demand for healthcare services. Weeks et al. demonstrate that healthcare providers can induce a demand for greater utilization of healthcare resources [2].

There are limited studies, however, that have explored the role of patient preferences on the utilization of healthcare resources. For instance, Tak et al. demonstrated that patients who are more engaged in their care are associated with increased healthcare utilization among hospitalized patients [3]. While these findings are provocative, there remains a poor understanding regarding the true impact of patient preferences on utilization of certain treatment options.

The surgical management of localized prostate cancer provides a pertinent preference-specific procedure to study patient demand for utilization of healthcare resources. The popularity of minimally invasive radical prostatectomy (MIRP) over open radical prostatectomy (ORP), particularly with the robotic surgical platform, has been anecdotally associated with direct-to-consumer advertising and market forces [5] rather than definitive clinical benefit [6–9]. Our recent work demonstrates heterogeneous dissemination of MIRP, with MIRP becoming the predominant form of surgery among civilian hospitals while ORP remains the most common surgical procedure in military institutions [10]. This dichotomy in the surgical approach for radical prostatectomy provides a unique opportunity to evaluate the presence of “patient-induced demand” for MIRP by examining a contemporary cohort of men in TRICARE, a health care program of the United States Department of Defense (DOD) that allows men with prostate cancer to receive care in either military or civilian facilities.

## Methods

### Aims

The aims of this study were to determine the predictors of the transference of care from one healthcare system to another and the receipt of MIRP.

### Data

The study utilized TRICARE insurance claims data (2006-2010) from the Military Health System Data Repository (MDR). TRICARE is the healthcare insurance program for uniformed service personnel, retirees and their dependents in the United States. TRICARE Prime beneficiaries receive comprehensive medical coverage through either the Direct Care System (DCS) at military facilities, or the Purchased Care System (PCS) at civilian facilities. Healthcare providers at the military facilities are salaried employees while those within the civilian facilities work at a mix of for-profit and non-profit civilian hospitals. Longitudinal follow up is available for patients while they are covered by TRICARE. We limited our analysis to TRICARE Prime for two reasons. First, TRICARE Prime enrollees are assigned a primary care manager (PCM), who manages their healthcare. Thus, they are less likely to receive care that is not in the purview of TRICARE coverage and the care provided would be better documented. Second, these plans have a low to no enrollment fee, no copayments and deductibles if care is provided within a military facility or if the patient was referred out through the military facilities or for those living outside a defined geographic catchment area of a military facility. Thus patients could opt for either a civilian or a military facility without cost being a barrier.

TRICARE is neither used to treat soldiers in combat zones nor provide care administered though Veterans Administration (VA) facilities. The TRICARE database, population being treated, mode of healthcare delivery and validation of the data elements have been described in prior literature [11–13].

### Study cohort, outcomes and covariates

We identified 5082 men diagnosed with prostate cancer (*International Classification of Disease, 9th Revision* [ICD-9]: 185) who underwent radical prostatectomy (RP; ICD-9: 60.5) from 2006 to 2010. Patients were dichotomized by the type of RP as ORP (ICD-9 60.5 only) or MIRP (ICD-9 60.5 + 54.21 and/or 17.42). Information was abstracted for the institution where a TRICARE beneficiary received his diagnosis of prostate cancer and subsequent surgery.

Men diagnosed at a military facility but who later underwent RP at a civilian facility were defined as having “transferred care”. Transference of care and receipt of MIRP were outcomes of interest in the primary and secondary analysis, respectively.

Demographics, Medicare eligibility, income (based on rank), comorbid condition and procedure type (MRP vs. ORP) were taken as patient level covariates while prostatectomy volume and availability of MIRP were taken as hospital level covariates.

### Primary analysis

For our primary analysis, we sought to determine the predictors for transferring care for surgery from a military to a civilian facility. We restricted our patient cohort to men residing within 40 miles of a military facility performing RP because those who live farther away can seek care at civilian facilities at little or no out of pocket costs. Therefore, our analytic cohort was comprised of patients who were arguably financially incentivized to remain within a military facility for the treatment. The adjusted model incorporated demographic information, including age, self-reported race/ethnicity, marital status, and military rank. To account for patient health status, we utilized the Centers for Medicare and Medicaid Services-Hierarchical Condition Category (CMS-HCC) model [14]. We also included the year of procedure to assess for differences across the study period. Hospitals were segregated by surgical volume into low (<24 RP annually) and high (≥24 RP annually) based on an a priori assumption that high volume centers perform at least two RP per month.

### Secondary analysis

To further evaluate whether the type of surgery (MIRP vs. ORP) may have influenced the decision to transfer health care systems, we created a second cohort comprised of TRICARE beneficiaries with prostate cancer specifically undergoing RP at a civilian facility representing 1026 men. This cohort was dichotomized into men who received their diagnosis and treatment at a civilian facility and those who received their diagnosis at a military facility and then transferred care to a civilian facility for the treatment, to determine if transferring care from military to civilian facilities was associated with a receipt of MIRP. Our adjusted model included the same clinical, demographic and hospital variables as in the primary analysis.

### Statistical analysis

We summarized patient and surgical characteristics for patients who underwent RP with descriptive statistics and multivariable logistic regression, comparing those who transferred care with those who did not transfer care (primary analysis) and comparing those who underwent ORP vs. MIRP at civilian facilities (secondary analysis). Chi square tests were used to compare categorical variables and ANOVA was used for continuous variables. We accounted for inter-hospital variations by clustering at the hospital level. Statistical analyses were performed using SAS 9.3 (SAS Institute, NC). All tests were two-sided and a *p*-value of <0.05 was considered statistically significant. The study protocol was reviewed and approved by our institutional review board. Permission to access data within MDR was obtained by a data use agreement between our institute and the Uniformed Services University of Health Sciences.

## Results

**Fig. 1:**
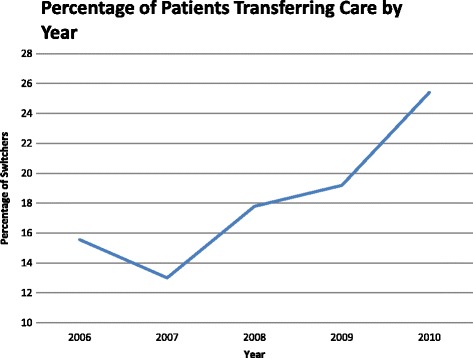
Proportion of TRICARE beneficiaries in the United States who, after diagnosis of prostate cancer at a military facility, transferred care to a civilian facility to undergo radical prostatectomy (*p* < 0.01)

Among the 1420 men in the primary analysis focused on men diagnosed with prostate cancer at military facilities, 247 (17.4%) transferred care from military to civilian facilities for their treatment (Table 1). Men who transferred care tended to be slightly younger (median age 57.8 vs. 59 years, *p* = 0.01), had a higher median HCC (0.39 vs. 0.37, *p* < 0.01), and were more likely Black when compared to men did not transfer (31.2% vs. 25.2% *p* < 0.01). Among men who transferred care to a civilian facility, 45.3% underwent MIRP, while among men who remained at military facilities, 13.7% underwent MIRP (*p* < 0.01). Significant differences were also noted by year with proportion of patients transferring care increasing from 15.6% in 2006 to 25.4% in 2010 (*p* < 0.01) (Fig. 1).

**Table 1:** Demographic and clinical characteristics of TRICARE beneficiaries in the United States who, after diagnosis of prostate cancer at a military facilities, transferred their care to a civilian facilities to undergo radical prostatectomy, compared to those who did not transfer care

Characteristic	Non-Transferers	Transferers	*p*-value
*n* (%)	1173 (82.6%)	247 (17.4%)	
Median Age at Procedure	59	57.8	
Mean Age at Procedure	58.48 (8.1)	57.11 (7.6)	0.01
Race			<0.01
White	754 (64.3%)	122 (49.4%)	
Black	295 (25.2%)	77 (31.2%)	
American Indian/Asian/Other	124 (10.6%)	13 (5.3%)	
Unknown	0 (0.0%)	35 (14.2%)	
Marital Status			0.09
Married	971 (82.8%)	191 (77.3%)	
Single	119 (10.1%)	36 (14.6%)	
Unknown	83 (7.1%)	20 (8.1%)	
Beneficiary category			0.98
Active duty	164 (14.0%)	35 (14.2%)	
Non-active duty	1009 (86.0%)	212 (85.8%)	
Medicare eligibility			0.06
Eligible	216 (18.4%)	33 (13.4%)	
Not eligible	957 (81.6%)	214 (86.6%)	
Sponsor rank			0.80
Enlisted	693 (59.1%)	139 (56.3%)	
Officer	388 (33.1%)	84 (34.0%)	
Unknown	92 (7.8%)	24 (9.7%)	
Procedure Type			<0.01
Open	1012 (82.3%)	135 (54.7%)	
Minimally Invasive	161 (13.7%)	112 (45.3%)	
Mean HCC	0.37 0.24	0.40 0.23	<0.01

In adjusted analysis, men who transferred their care had higher adjusted odds of identifying with a minority (non-White) race [odds ratio (OR): 1.96, 95% Confidence Interval (95% CI): 1.34-2.87, *p* < 0.01], undergoing MIRP (OR: 7.83, 95% CI: 3.30-18.62, *p* < 0.01), and being diagnosed at a low volume center for RP (<24 RP annually) (OR: 6.10, 95% CI: 2.20-16.95, *p* < 0.01). (Table 2 **)** Less healthy individuals were also more likely to seek care at civilian institutions [HCC: (OR: 2.88 95% CI: 1.38-5.98, *p* < 0.01)]. In contrast, active duty status was associated with lower likelihood of transferring care (OR: 0.54, 95% CI: 0.32-0.90, *p* = 0.05).

**Table 2:** Risk-adjusted predictors of transferring care from a military to a civilian facility among TRICARE beneficiaries in the United States

Predictors	OR	95%CI		*p*-value
Age at procedure	0.98	0.94	1.01	0.11
Race				
White	Reference			
Non-white race	1.96	1.34	2.87	<0.01
Marital Status				
Single	Reference			
Married	0.69	0.46	1.03	0.07
Beneficiary category				
Non-active duty	Reference			
Active duty	0.54	0.32	0.90	0.05
Medicare eligibility				
In-eligible	Reference			
Eligible	0.98	0.38	2.53	0.97
Income (based on rank)				
Low	Reference			
High	1.50	0.41	5.43	0.54
Rank				
Officer	Reference			
Enlisted	0.94	0.28	3.11	0.92
HCC	2.88	1.38	5.98	<0.01
Procedure Type				
Open	Reference			
Minimally invasive	7.84	3.30	18.63	<0.01
Volume of center of diagnosis				
High volume	Reference			
Low volume	6.10	2.20	16.95	<0.01
MIRP availability at center of diagnosis				
Available				
Not available	1.61	0.93	2.78	0.09

In the secondary analysis, among the 1026 TRICARE beneficiaries who underwent surgery in a civilian facility, 247 (24.1%) transferred care from a military facility. Compared to patients initially diagnosed at civilian facilities, transferers from military facilities were more likely to be non-White as (49.4% versus 38.6%, *p* < 0.01) and less healthy [median HCC:(0.39 versus 0.33, *p* < 0.01)] (Appendix 1).

The independent predictors of undergoing MIRP in the secondary analysis are presented in Table 3. Patients undergoing MIRP had higher risk adjusted odds of being transfers from a military facility (OR: 1.51, 95% CI: 1.03-2.22, *p* = 0.04). The odds of undergoing MIRP were also inversely associated with increasing HCC (OR: 0.28, 95% CI: 0.09-0.83] per 1.0 unit increase, *p* = 0.02). MIRP was also more likely to be performed in 2010 compared to 2006 (OR: 33.33, 95% CI: 20.00-50.00, *p* < 0.01).

**Table 3:** Risk-adjusted predictors of undergoing minimally invasive radical prostatectomy among TRICARE beneficiaries undergoing prostate cancer surgery in the United States

Predictors	Probability of receiving minimally invasive procedure			
	OR	95%CI		*p*-value
Age at procedure	1.01	0.99	1.03	0.64
Race				
White	Reference			
Non-white	0.93	0.67	1.30	0.68
Marital Status				
Single	Reference			
Married	1.05	0.65	1.70	0.85
Rank				
Officer				
Enlisted	0.82	0.58	1.18	0.29
HCC	0.28	0.09	0.83	0.02
Transferring Care Status				
No	Reference			
Yes	1.51	1.02	2.22	0.04
Year of procedure				
2006	Reference			
2010	33.33	20.00	50.00	<0.01

## Discussion

In this study of TRICARE beneficiaries diagnosed with prostate cancer who have access to care at military and civilian facilities, we found evidence supporting the premise that “patient-induced demand” drives the utilization of MIRP. Our study shows that a substantial proportion of patients (17.4%) transferred care from military to civilian facilities in order to undergo RP, despite logistic incentives for staying within the military healthcare system for treatment. Both analyses demonstrate that the men who transferred care were most strongly associated with undergoing MIRP. These findings support the hypothesis that patient preferences may represent an important factor for inducing the demand for utilization of healthcare services for surgical intervention.

The main strength of this study is the uniqueness of the patient population. TRICARE patients are universally insured under the same TRICARE Prime benefit plan. This minimizes the possibility of confounding by insurance status and different types of insurance coverage. Secondly, these patients not only had the logistical advantage of living close to a military facility (within 40 miles) that offered radical prostatectomy but also the financial incentive of receiving care completely free of charge by remaining at the military facility. Despite these benefits, a sizable proportion (nearly one-fifth) of these patients chose to transfer care to civilian facilities. Since out-of-pocket costs at civilian facilities would act as a deterrent for transferring care, this is further evidence in support of the relationship between patient preferences and the demand for healthcare utilization.

To the best of our knowledge, this is the first study to investigate the impact of prostate cancer patient preferences as the driving force for the utilization of a specific surgical intervention. The possibility for patient-induced demand for MIRP among men with prostate cancer was previously implied by Chang et al. [15] and Makarov et al. [16] who reported that the availability of robotic technology increased the volume of RP for individual surgeons and hospitals, respectively. Previous studies among men with prostate cancer have demonstrated patient preferences for various treatment options for prostate cancer (e.g. active surveillance, surgery, radiation, or hormonal therapy) [17–19], cosmetic results of different types of prostatectomies [20] and on functional outcomes (erectile function and continence) [21]. Our results expand on this pre-existing body of work, and demonstrate that patient preferences indeed induce a demand for greater utilization of healthcare services, which could feasibly impact the overall burden of disease.

Our secondary analysis studied civilian facilities to which TRICARE beneficiaries with prostate cancer transferred care from a military facility, to identify possible motivations for transferring care. We found that transferring care from a military facility was strongly associated with undergoing MIRP. These findings suggest that even within the civilian facilities, patients transferring care are more likely to undergo MIRP compared to patients who were initially diagnosed at and remained at the civilian facilities for surgery. This may imply that the demand for greater utilization of a particular type of care may be driven by a subset of patients who are highly motivated, and proactive in their healthcare, having strong preferences for a particular treatment option. Our findings are consistent with the findings of Tak et al.’s study, which demonstrated that patient preferences regarding participation in medical decision making was associated with greater resource utilization (i.e. longer length of stay and higher total hospitalization costs) [3].

The present study findings suggest that promoting new technology, even without clear scientific evidence showing advantages compared to standard options, may stimulate patient-induced demand thereby attracting new patients and increasing volume. Our findings are consistent with previous work done on the impact of direct-to-consumer advertising (DTCA) on healthcare utilization in the field of pharmacy [22, 23]. In an article titled, “*Just What the Patient Ordered? Direct-to-Consumer Advertising and the Demand for Pharmaceutical Products*” [22], the author demonstrates that DTCA plays a significant role in the expansion of market of a certain category of drugs. Bradford et al. evaluated the association of DTCA for osteoarthritis drugs with physicians’ prescribing behavior [23]. Their work elucidates that DTCA not only increased the likelihood of the said drug being prescribed but also resulted in larger numbers of patients seen for osteoarthritis. These findings suggest that patient preferences not only impact the utilization of a certain treatment option but also increase the utilization of healthcare services as a whole. Marketing to patients therefore may also explain in part the widespread diffusion of robotic technology for MIRP in the United States, even in the absence of irrefutable scientific data showing its benefits over ORP. Our work indicates the possible magnitude of this impact and demonstrates how marketing might lead to greater utilization of a novel surgical technology.

The preference for MIRP among patients is likely borne out of a fundamental patient desire for optimal care and outcomes, although Level I evidence has shown no appreciable difference between surgical approaches [24]. In contrast, patient awareness that higher surgical volume has been consistently associated with improved outcomes following prostate cancer surgery [25] likely explains why diagnosis of prostate cancer at a military facility with a low volume of radical prostatectomies was a strong predictor of patient request for transfer of care to a civilian facility (Table 1). Additional investigation, however, is warranted to better understand our finding that non-white men and less healthy men were also more likely to transfer care from a military to a civilian facility for definitive surgical management of prostate cancer.

The percentage of patients transferring care from a military to a civilian facility increased steadily throughout the study period (Fig. 1). This finding is likely associated with the disproportionately faster and more widespread adoption of MIRP among civilian facilities compared to military facilities. These differences in adoption rates of MIRP have been attributed to differences in the financial structure of these two health care systems [10]. The military healthcare system has a salaried system of compensation for healthcare providers and funds may be allocated to preventive care. On the contrary, most civilian facilities have a fee-for-service model and healthcare delivery is reliant on generation of sufficient revenue. This is a possible incentive for adopting new technology to attract new patients and increase volume to maximize revenue [26].

Given the association between patient preferences and receipt of MIRP, some interesting questions arise. First, what is the driving force behind these patient preferences, given that the scientific literature on the benefits and risks of MIRP over ORP is mixed? [6–8, 24] Second, how can these preferences be changed to align with current evidence and cost considerations? The answer to the first question may be attributed to the increased use of internet for health-related information [27] and online information is biased towards robot-assisted radical prostatectomy [28]. Hajdenberg et al. evaluated the quality of information on robot-assisted radical prostatectomy present on the internet, and found that websites that advertise robot-assisted radical prostatectomy were more appealing than those that do not specifically advertise the procedure and instead simply provide information. Mirkin et al. in a similar effort evaluated the credibility of the information present on the internet regarding robot-assisted radical prostatectomy [29], and demonstrated that many websites claim benefits, not supported by evidence, with as many as 42% of websites failing to mention risks associated with the procedure. For the second question, the possible solutions include the need for greater regulatory control over advertisement and creation of well-balanced patient education material, so patients can make more informed decisions regarding their treatment preferences.

### Limitations

This study has some limitations that need to be considered when drawing conclusion based on these results. First, we used the TRICARE database, which relied on medical claims and encounter data. Like other administrative datasets, we cannot account for some nuanced clinical characteristics that may have an impact on the association that we demonstrated, such as oncologic characteristics. However, if RP was clinically indicated, then it is generally safe to assume that all patients identified had localized prostate cancer amenable for surgical intervention, and other factors such as PSA levels, Gleason score or TNM staging may not influence results significantly [30]. Second, there exists the possibility of coding errors leading to misclassification bias. However, TRICARE has many structural similarities with Medicare, a similar database, which has been used widely for surgical outcome studies [31]. Third, we were not able to reliably account for the availability of a robot at a particular facility. Adoption of clinical innovation for the maximization of hospital revenue could be a major driver for more rapid adoption of MIRP in civilian facilities. However, it must be considered that civilian facilities are much more likely to have earlier robotic surgery capability than military facilities. The availability of specialized equipment and trained personnel are as likely (or more likely) to contribute to the more rapid and widespread adoption of MIRP in civilian facilities [10].

## Conclusions

This is the first study to quantify the potential impact of patient preferences on the utilization of surgical technology using administrative data from a large population with universal insurance coverage. Low prostatectomy volume and unavailability of MIRP at the hospital of diagnosis were strong predictors of transferring care to civilian facilities despite significant financial incentives at military facilities. These findings suggest that patients’ preferences induce a demand for greater utilization of MIRP. This phenomenon is particularly important in our current resource-constrained healthcare environment. Interventions aimed at cost containment, such as greater regulatory control for the advertisement of medical technology not supported by evidence, and development of balanced patient educational material, should be considered. Further work needs to focus on factors affecting patient preferences for certain healthcare technologies and treatment options, and the development of interventions to tailor these preferences according to the latest scientific evidence and cost considerations.

## Appendix 1

**Table 4:** Demographic and clinical characteristics of TRICARE beneficiaries who were diagnosed and treated for prostate cancer at civilian facilities compared to those who transferred care from military facilities to civilian facilities after diagnosis

	Civilian facility patients	Military Facility patients Transferring Care	
Characteristic *n* (%)	779 (75.9%)	247 (24.1%)	*p*-value
Mean Age at Procedure	58.2	57.8	
Race			<0.01
White	478 (61.4%)	122 (49.4%)	
Non-white	301 (38.6%)	122 (49.4%)	
Marital Status			0.08
Married	651 (83.6%)	191 (77.3%)	
Single	80 (10.3%)	36 (14.6%)	
Unknown	48 (6.2%)	20 (8.1%)	
Sponsor rank			<0.01
Enlisted	504 (64.7%)	139 (56.3%)	
Officer	216 (27.7%)	84 (34.0%)	
Unknown	59 (7.6%)	24 (9.7%)	
Procedure Type			0.18
Open	463 (59.4%)	135 (54.7%)	
Minimally Invasive	316 (40.5%)	112 (45.3%)	
Year of Procedure			0.32
2006	144 (18.5%)	56 (22.7%)	
2007	133 (17.1%)	42 (17.0%)	
2008	177 (22.7%)	50 (20.2%)	
2009	199 (25.6%)	52 (21.0%)	
2010	126 (16.2%)	47 (19.0%)	
Median HCC	0.33	0.39	

## Abbreviations

DCS: Direct Care System (Military Healthcare Facilities)
DoD: Department of Defense
MDR: Military Health System Data Repository
MIRP: Minimally Invasive Radical Prostatectomy
ORP: Open Radical Prostatectomy
PCS: Purchased Care System (Civilian Healthcare Facilities)
